# Overestimating resistance in field testing of malaria parasites: simple methods for estimating high EC_50 _values using a Bayesian approach

**DOI:** 10.1186/1475-2875-6-4

**Published:** 2007-01-17

**Authors:** Kasia Stepniewska, Kesinee Chotivanich, Alan Brockman, Nicholas PJ Day, Nicholas J White

**Affiliations:** 1 Faculty of Tropical Medicine, Mahidol University, 420/6 Rajvithi Rd., Bangkok 10400, Thailand; 2 Centre for Tropical Medicine and Vaccinology, Churchill Hospital, Oxford, UK; 3 Shoklo Malaria Research Unit, Mae Sot, Thailand

## Abstract

Conventional methods of assessing in-vitro antimalarial drug-concentration effect relationships in field testing of fresh isolates assess each parasite isolate individually. This leads to systematic overestimation of EC_50 _values for the most resistant isolates, and thus overestimation of the degree of resistance. In antimalarial drug-susceptibility studies conducted on the north-western border of Thailand the overestimation of EC_50 _for the most resistant isolate ranged from 15% for artesunate to 43% for mefloquine. If isolates cannot be stored for re-testing, more accurate estimations of the degree of resistance can be obtained using a Bayesian approach to data analysis which is described here.

## Background

The development of resistance to antimalarial drugs poses one of the greatest threats to malaria control and is the main cause of recent increases in malaria morbidity and mortality. The precise quantitation of resistance is therefore of prime importance. Initially the only way of assessing resistance to antimalarials was by inference from clinical treatment failures. Occasionally these were supported by measurement of antimalarial drug concentrations in the patients' blood. Once methods of culturing *Plasmodium falciparum *became established, *in vitro *methods for measuring the effect of the antimalarial drug directly on malaria parasite were developed [1]. This allowed resistance (i.e. reduced susceptibility) to be differentiated from poor adherence or unusual pharmacokinetics as the cause of treatment failure.

In *in vitro *susceptibility tests, blood samples from malaria patients are obtained and the infecting malaria parasites are cultured ex-vivo in the presence of stepwise increases in the concentrations of antimalarial drugs. Some methods call for adaptation of parasites to culture first, while others put blood directly from patients into the test system. Field testing, where blood is taken and malaria parasites are cultured directly in 96-well plastic plates pre-dosed with antimalarial at different concentrations, is now widely used. These freshly obtained parasites are usually not cryo-preserved and so there is only the one opportunity to assess the parasite drug susceptibility.

### Problems with estimating EC_50 _for resistant isolates

**Figure 1:**
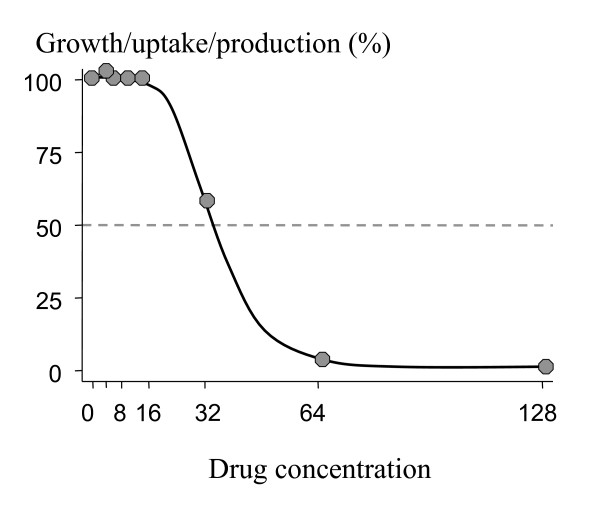
Standard dose-response curve in antimalarial drug susceptibility testing (arbitrary units). In this case the response or effect is inhibition of growth, uptake, or synthesis.

The three most commonly used methods of *in vitro *susceptibility testing of malaria parasites are (i) the micro test which assesses inhibition of parasite growth to the schizont stage microscopically, (ii) the radioisotope test which measures uptake of ^3^H-hypoxanthine, and (iii) ELISA based methods which measure the production of lactate dehydrogenase or histidine rich protein by the parasite [2,3]. Results of *in vitro *tests are expressed as the percentage parasite growth/viability plotted against the antimalarial drug concentration (or log concentration) to give a dose-response (concentration-effect) curve. The range of drug concentrations used are chosen because they are thought *a priori *to reflect the likely range of parasite susceptibilities and they are usually prepared as serial doubling dilutions The resulting dose-response curve is usually sigmoid (Figure 1) and can be described by a number of parameters: minimum growth/uptake/production, maximum growth/uptake/production, the slope, and importantly the midway point (EC_50_) describing the concentration of drug which is required for 50% of the maximum inhibitory effect in the test system. These variables can be read directly from the plot, assessed by probit analysis, or they require fitting of a statistical model to data and then derivation from the equation of the model. A commonly used model is the sigmoid Emax model:

**E(C) **= **E**_**max **_**- (E**_**max **_**- E**_**min**_**)C**^***γ***^**/(C**^***γ***^**+EC**_**50**_^***γ***^**)**

where E_max _is the maximum effect (e.g. minimum growth), E_min _is the minimum effect (maximum growth), C is the concentration of drug, *γ *is the slope of the linear part of the curve, and EC_50 _is defined as before. Parameter E_min _sometimes is set to 0 to avoid overparameterization.

**Figure 2:**
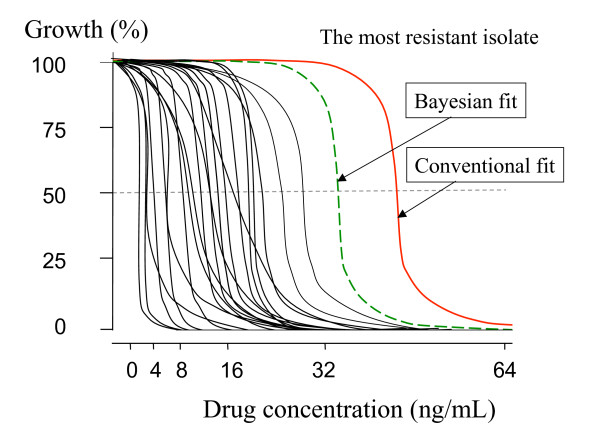
Illustrative example of a distribution of antimalarial susceptibility concentration-growth inhibition curves fitted using standard models. The most resistant isolate's curve fit is poor as inhibition was obtained only with the highest concentration tested (64 ng/mL), and so the EC_50 _derived using standard curve fitting lies at the mid-point (48 ng/mL) between the two highest concentrations tested (32 and 64 ng/mL). The Bayesian approach (dotted line) provides a lower estimate closer to the rest of the population.

**Figure 3:**
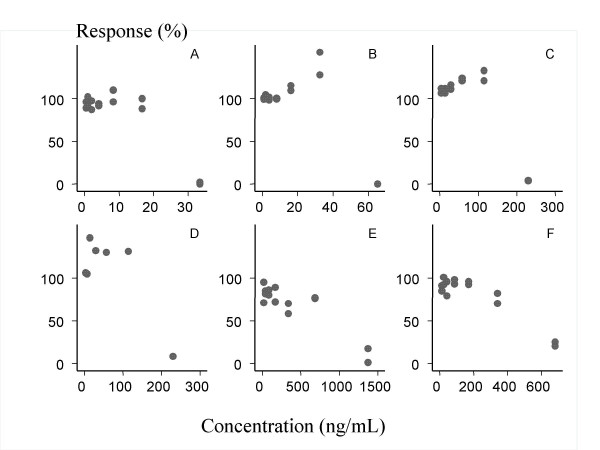
Dose-Response relationship; real data from ^3^hypoxanthine uptake inhibition in-vitro susceptibility tests for the most resistant isolates in the series (6) for (A) Artesunate, (B) Halofantrine, (C) Mefloquine, (D) Mefloquine, (E) Chloroquine, (F) Chloroquine.

The most resistant isolate(s), by definition, represents the extreme value in one tail of a distribution of values. These values lie at the lower end of the dilution range (Figure 2). As concentrations are taken usually as serial dilutions, there are less measurements of drug effect at this end of range (because most of the concentrations in the test system have no effect). The relatively large intervals between the highest drug concentrations in the test system mean that often the middle range of inhibitory effect is not observed at all; for one dilution there may be no (or very little) drug effect and for the next dilution the maximum (or close to maximum) effect is measured (Figures 1 to 3). If the distribution of all EC_50 _values in the parasite population is unimodal (and this is a critical point) then, given that the EC_50 _lies at an unknown point between two values (two dilutions), the prior probability suggests that the true value lies closer to the median value of the population than to the more extreme value.

But the fit, and thus estimated EC_50 _value, is based only on the observations for that isolate. It is independent of the distribution of values from other isolates in the series and, in case of the sigmoid model, a symmetrical curve is fitted. The fit is poor but the midpoint value will lie close to half way between the two concentrations tested (Figure 2).

Standard fitting procedures, therefore, systematically overestimate EC_50 _values for the most resistant parasites. Moreover, when curve fitting is attempted for resistant isolates and only responses close to 100% or 0% are observed, often either computational problems occur and the curve cannot be fitted at all, or the curve is fitted but the standard errors of the estimates are large. In this case, a priori information about the population could be very useful in planning the future experiments and deciding where the EC_50 _is expected to be and where the concentrations measurements should be taken in order to pinpoint the 50% response. In this report a Bayesian approach to the estimation of EC_50 _in the outlying most resistant isolates together with worked examples from field data is presented.

## Methods

These suggestions apply to field methods of assessing *in vitro *susceptibility to malaria parasites where the inhibitory values (EC_50_, EC_90_, etc.) are extrapolated from observations over a preset concentration range. This range is set based on the earlier experience with field testing. The criteria for "goodness of fit" have not been standardized and, therefore, vary between investigators. The variation will affect the results, but this important issue is not discussed here.

### Estimating EC_50 _based on the population estimates

If, from the *in vitro *drug susceptibility experiment, it is known that the EC_50 _lies in the interval (a to b), where b = 2a if doubling solutions were used, the Bayesian estimate of EC_50 _in this interval can be constructed in the following way:

1. estimate distribution of EC_50 _values for this batch of isolates (using 'good' estimates coming from 'good' fits). This involves estimating EC_50 _for each isolate.

2. using distribution of EC_50_, calculate median value of EC_50 _in the interval (a,b)

3. this summary measure value is the Bayesian estimate of EC_50 _in the interval (a,b).

Characterization of the distribution of EC_50 _values is required, and this is not always possible, but if the EC_50 _values can be shown to have a normal or lognormal distribution or can be transformed to normal using Box-Cox transformation [4], then the estimation of the corrected EC_50 _value can be done using a pocket calculator, as described below. The corrections suggested apply to continuous distributions and are not appropriate for clearly discontinuous distributions (for example, those seen where one or more parasite isolates contain the cytochrome b mutations which confer high level atovaquone resistance in *P. falciparum*).

### Normal distribution

Probability density f(x) of a normally distributed random variable x is given by the expression

f(x) = 1/(*σ*2
 MathType@MTEF@5@5@+=feaafiart1ev1aaatCvAUfKttLearuWrP9MDH5MBPbIqV92AaeXatLxBI9gBaebbnrfifHhDYfgasaacH8akY=wiFfYdH8Gipec8Eeeu0xXdbba9frFj0=OqFfea0dXdd9vqai=hGuQ8kuc9pgc9s8qqaq=dirpe0xb9q8qiLsFr0=vr0=vr0dc8meaabaqaciaacaGaaeqabaqabeGadaaakeaadaGcaaqaaiabikdaYaWcbeaaaaa@2DB9@*π*)·exp(-(x-*μ*)^2^/2*σ*^2^)

where exp(z) = e^z^, *μ *is the expectation or mean value of x and *σ *is the standard deviation of x.

The median value of x on the interval (a, b) is calculated (see Appendix II) as:

Median (x) = Φ^-1 ^((Φ((a-*μ*)/*σ*) + Φ((b-*μ*)/*σ*))/2)·*σ *+ *μ *    (1)

where Φ(t) = ∫−∞tf(z)
 MathType@MTEF@5@5@+=feaafiart1ev1aaatCvAUfKttLearuWrP9MDH5MBPbIqV92AaeXatLxBI9gBaebbnrfifHhDYfgasaacH8akY=wiFfYdH8Gipec8Eeeu0xXdbba9frFj0=OqFfea0dXdd9vqai=hGuQ8kuc9pgc9s8qqaq=dirpe0xb9q8qiLsFr0=vr0=vr0dc8meaabaqaciaacaGaaeqabaqabeGadaaakeaadaWdXaqaaiabbAgaMjabcIcaOiabbQha6jabcMcaPaWcbaGaeyOeI0IaeyOhIukabaGaeeiDaqhaniabgUIiYdaaaa@3722@ dz and z is a standard normal distribution N(1,0). Values of Φ(z) are tabulated and can be found in any book with statistical tables.

### Lognormal distribution

A random variable y is log-normally distributed if x = log(y) is normally distributed with log denoting the natural logarithm. The general formula for the probability density function of the lognormal distribution is

f(y) = 1/((y-*θ*)*σ*2
 MathType@MTEF@5@5@+=feaafiart1ev1aaatCvAUfKttLearuWrP9MDH5MBPbIqV92AaeXatLxBI9gBaebbnrfifHhDYfgasaacH8akY=wiFfYdH8Gipec8Eeeu0xXdbba9frFj0=OqFfea0dXdd9vqai=hGuQ8kuc9pgc9s8qqaq=dirpe0xb9q8qiLsFr0=vr0=vr0dc8meaabaqaciaacaGaaeqabaqabeGadaaakeaadaGcaaqaaiabikdaYaWcbeaaaaa@2DB9@*π*)·exp(-(log(y-*θ*)/m)^2^/2*σ*^2^)

where *σ *is the shape parameter, *θ *is the location parameter and m is the scale parameter (equal to median). The calculation of the median value in the interval (a,b) may be performed using the log-transformed data. Firstly, the median of transformed x needs to be found in interval (log(a), log(b)). As transformed x is normally distributed, methods described in the paragraph above apply and equation (1) can be used. Then the median needs to be back-transformed (using the exponent function) to the original scale. Because of monotonic transformation, the median of transformed x in interval (log(a), log(b)) corresponds to the median of x in the interval (a,b)

Median (x) = exp (Φ^-1 ^((Φ((log(a)-*μ*)/*σ*) + Φ((log(b)-*μ*)/*σ*))/2)·*σ *+ *μ*)     (2)

### Box -Cox family of transformations

The Box-Cox transformation is defined as:

T(y) = (y^*λ*^-1)/*λ*

where y is the random variable and *λ *is the transformation parameter. For *λ *= 0, the natural log of the data is taken instead of using the above formula. Similarly as before, if T(y) has an median value m in interval (T(a), T(b)) then median of y in interval (a,b) is m^1/*λ*^.

### Simple method using computer simulation

An easier way to obtain the Bayesian estimate of the EC_50_, for data where the effect goes from 100% to 0% over a single dilution, which does not require any equations, is to use computer simulations. They can be done in Excel^®^, or with any statistical software. Once the distribution of EC_50 _is established, a large number of points (for example 10^6^) from this distribution can be readily generated and then the estimate of the EC_50 _for a resistant isolate can simply be found as a median value for all data points which are in the interval (a,b) between the two dilutions.

### Data

The data used to illustrate these issues come from the Shoklo Malaria Research Unit (SMRU) in Thailand. They are in-vitro susceptibility data of malaria parasites isolated from patients recruited in two camps for displaced persons of the Karen ethnic minority situated in an area of forested hills on the north-western border of Thailand. Antimalarial drug susceptibility data were obtained using the hypoxanthine uptake inhibition assay and have been described and analysed elsewhere [5]. In total, 268 fresh isolates of *P. falciparum *from primary infections were assayed for *in vitro *drug susceptibilities to a wide range of antimalarials including chloroquine diphosphate, quinine citrate, mefloquine hydrochloride, halofantrine hydrochloride, artesunate, dihydroartemisinin, artemether, lumefantrine, and atovaquone. To illustrate this particular issue we selected data for four drugs; mefloquine, chloroquine, halofantrine and artesunate.

### Statistical Analysis

For each of the examined drugs, the distribution of EC_50 _values was estimated in the following way:

1. alll isolates which had at least one inhibitory response value between 30 and 70% of the maximum response were identified and selected as this was considered a priori to be a minimum requirement for the data points to give stable estimates

2. the E_max _model using Stata^® ^software (StataCorp. 2005, ver 9) was fitted to each of the selected isolates

3. isolates for which the model did not reach convergence were excluded

4. isolates which had negative estimates of EC_50 _were excluded

5. the distribution of EC_50 _values in the remaining isolates (N) was examined

6. If possible, EC_50 _values were transformed (logarithmic or Box-Cox transformation) to normality. Normality of the distributions was tested using the Kolmogorov-Smirnov test.

For each of the isolates which were most resistant to mefloquine, halofantrine, chloroquine and artesunate the EC_50 _values were estimated using three methods: (a) by fitting a standard 3-parameter sigmoid curve using WinNonlin^® ^(Pharsight, ver 4.1); (b) by fitting the 3-parameter sigmoid curve using Gibbs sampling using WinBUGS^®^; (c) by the "Bayesian" method based on the population distribution of EC_50 _values proposed in this paper (equation 1).

The standard sigmoid model (a) was fitted using Nelder-Mead minimisation, with no specified boundaries for parameters and WinNonlin^® ^generated starting values. In the Gibbs sampling estimation (b) the distribution of EC_50 _values was estimated as described above, while non-informative uniform priors with appropriate bounds were used for other parameters:

Emax ~ uniform(0.5,1.5)

*γ *~ uniform(1,100)

The effect of the bounds of parameter *γ *on estimates of EC_50 _were also examined by restricting the upper bound to 30, and then to 50. Parameter estimates were summarized as median and 95% range of the posterior estimates over 90,000 iterations.

## Results

The measured responses of isolates most resistant to mefloquine (two series), halofantrine, chloroquine (two series) and artesunate are presented in Figure 3.

Table 1 describes estimated distributions of the EC_50 _values for each drug. All of them could be assumed to be log-normally distributed. Overall, after the selection described in points 1, 3 and 4, about half of the isolates contributed to the estimation.

**Table 1:** Estimated distribution of EC_50 _values

*Drug*	**Mean**^1^	**SD**^1^	**N (%)**^2^	**P-value**^3^
Artesunate	0.75	0.888	89 (47)	0.074
Halofantrine	1.84	0.979	85 (44)	0.100
Mefloquine	3.68	0.633	124 (58)	0.366
Chloroquine	4.9	0.680	117 (64)	0.076

^1 ^mean and SD after the log-transformation

^2 ^number of isolates which contributed to the estimation of the distribution

^3 ^Kolmogorov-Smirnov test for normality

Table 2 lists parameter estimates obtained from the WinNonlin analysis (a) for the resistant isolates. Only for artesunate and chloroquine do the estimates have reasonable precision, for the other drugs the standard errors (CV%) are very large. This means that the model does not estimate the parameters accurately. In fact, when refitting the model with different starting values, different estimates of EC_50 _(within ± 10% of the quoted value) were obtained each time (data not shown). In Table 3, corresponding parameter estimates obtained from fitting the three-parameter sigmoid curve using the Gibbs sampling method (b) are listed. Obviously the slope of the concentration-effect relationship is a critical determinant of EC_50 _but the EC_50 _estimates were relatively insensitive to these prior slope estimates (*γ*); change of the upper bounds for parameter *γ *did not affect substantially the estimated values of parameters whereas slight increases in the EC_50 _estimates (<10%) were observed with lower bound changes (Table 4).

**Table 2:** Parameter estimates obtained from fitting the 3-parameter sigmoid curve to the most resistant isolates using standard individual data analysis (fitted using WinNonlin^®^).

*Isolate*	*Parameter estimates (% CV)*			
	**EC**_**50**_	**γ**	**Emax**	**EC**_50_*
Artesunate (A)	23.3(22)	12.5(65)	0.95 (2)	23.4 (12)
Halofantrine (B)	51.8(51564)	27.9 (225370)	1.10 (5)	50.1 (37)
Mefloquine (C)	195 (4160)	21.1 (25309)	1.15 (2)	175 (17)
Mefloquine (D)	197 (2014)	17.1 (12967)	1.25 (6)	178 (52)
Chloroquine (E)	1065 (18)	7.98 (68)	0.79 (4.2)	1065 (18)
Chloroquine (F)	536 (6)	3.5 (18)	0.92 (2.4)	536 (6)

* – estimates obtained by fitting model with boundary (-10,10) for **γ**

**Table 3:** Parameter estimates obtained from fitting the 3-parameter sigmoid curve to the data presented in the example using a Bayesian approach (Gibbs sampling) (fitted using WinBUGS^®^)

*Isolate*	*Posterior Parameter Estimates – median (95% range)*		
	**EC**_**50**_	***γ***	**Emax**
Artesunate (A)	21.3 (17.5 – 30.5)	59.7 (14.0 – 98.1)	0.95 (0.91 – 0.99)
Halofantrine (B)	43.7 (34.8 – 61.0)	61.3 (15.3 – 98.0)	1.09(1.00 – 1.19)
Mefloquine (C)	154 (123 – 215)	61.8 (16.2 – 98.2)	1.5(1.10 – 1.20)
Mefloquine (D)	150 (119 – 217)	57.0 (9.85 – 97.9)	1.25 (1.09 – 1.40)
Chloroquine (E)	873 (710 – 1294)	42.6 (6.2 – 78.2)	0.78 (0.71 – 0.85)
Chloroquine (F)	540 (461 – 673)	3.75 (2.52 – 55.2)	0.92 (0.88 – 0.97)

**Table 4:** Estimates of EC_50 _obtained from fitting the 3-parameter sigmoid curve to the data presented in the Example using WinBUGS, with upper boundary of *γ *set to 30 or 50.

*Isolate*	*Posterior Estimate of ***EC**_**50 **_– *median (95% range)*	
	***γ *= 30**	***γ *= 50**
Artesunate (A)	22.2 (18.6 – 28.2)	21.9 (18.0 – 29.8)
Halofantrine (B)	46.5 (37.3 – 60.0)	45.2 (36.0 – 60.0)
Mefloquine (C)	165 (134 – 205)	161 (128 – 210)
Mefloquine (D)	157 (123 – 212)	153 (121 – 215)
Chloroquine (E)	913 (731 – 1260)	888 (719 – 1282)
Chloroquine (F)	539 (465 – 649)	540 (462 – 660)

### Calculation of the Bayesian EC_50_

#### Halofantrine

In total 197 isolates were tested, of which 85 satisfied the criteria (see above, points 1–5). EC_50 _values estimated in these samples were found to have a lognormal distribution (p = 0.100, Kolmogorov-Smirnov test) with mean of 1.84 and standard deviation of 0.979; i.e. log(EC_50_) ~ N(1.84, 0.979) (Table 1).

From the equation (2) the EC_50 _was calculated as 41.2 ng/mL in the highest concentration interval (32.6 – 65.32 ng/mL).

#### Artesunate

Of 191 isolates, 89 satisfied the criteria and for them log(EC_50_) ~ N(0.75, 0.888), p = 0.074 (Table 1) In the highest concentration interval (16.73–33.46 ng/mL) an EC_50 _of 20.3 ng/mL is obtained.

#### Mefloquine

Out of 216 isolates, 124 satisfied the prespecified criteria and for these the EC_50 _values have a log-normal distribution (p = 0.366), log(EC_50_) ~ N(3.68, 0.633) (Table 1). In the highest concentration interval (115–230 ng/mL) an EC_50 _of 137.8 ng/mL was calculated.

#### Chloroquine

Out of 183 samples, 117 satisfied the criteria and for them log(EC_50_) ~ N(4.9, 0.680), p = 0.076 (Table 1). In the highest concentration interval (341.5–683 ng/mL) EC_50 _of 420.4 ng/mL was obtained.

In Table 5 Bayesian EC_50 _estimates (c) are compared with the standard estimates obtained using WinNonlin^® ^(b). The standard method oversestimated the EC_50 _of the most resistant isolate by between 15% (artesunate) and 43% (mefloquine).

**Table 5:** Methodological differences in the estimation of antimalarial resistance; the highest EC_50 _(ng/mL) (Brockman *et al*, 2000)

*Isolate*	*Range of concentrations tested (ng/mL)*	*EC*_*50 *_*Standard estimate*	*EC*_*50 *_*"Bayesian" estimate*	*Overestimation by standard method (%)*
Artesunate (A)	0.52 – 33.46	23.4	20.3	15%
Halofantrine (B)	1.02 – 65.32	51.8	41.2	26%
Mefloquine (C)	3.59 – 230	195	138	41%
Mefloquine (D)	3.59 – 230	197	138	43%
Chloroquine (E)	21.44 – 1,372	1065	801	32%
Chloroquine (F)	21.44 – 683	536	420	32%

## Discussion

Antimalarial drug resistance is widely monitored using in-vitro susceptibility testing. There are sentinel sites throughout the malaria affected world monitoring for drug resistance. A variety of methods have been developed and the results have provided valuable information in the assessment and mapping of antimalarial drug resistance [7]. Evaluation of stored isolates in reference centres allows proper standardisation of methodologies and repeated tests on single isolates. But most of this testing in the field is a "one-off" microtest on freshly obtained blood samples. When antimalarial drug susceptibility tests are reported the highest observed values observed are naturally of greatest interest as they may represent emerging drug resistance. Drug regimens should aim to cure all infections, and thus provide concentrations exceeding the inhibitory concentrations for the most resistant prevalent parasites. If only a single concentration range is evaluated in an in-vitro susceptibility assay using serial dilutions then, by definition, the true EC_50 _of the most resistant isolates must lie above or between the largest concentration differences tested. Thus, unless the parasites are retested with a higher concentration range (which they usually cannot be), the precision of the estimated EC50 or EC90 value of the most resistant isolate will usually be the poorest of all the isolates assayed. Furthermore as curve fitting or probit analysis takes no account of other isolates in the series tested, then if there are two adjacent points with extremely different values (often zero and 100% inhibition), a curve will be fitted as lying symmetrically between the two. The EC_50 _will be assessed as lying close to the mid-point between the two concentrations (Figure 2). But if the parasites can be shown to derive from a single distribution of susceptibilities, and this proviso is critical, the prior probability is that the true EC_50 _value lies closer to the population mean value. Thus resistance is systematically overestimated. A better estimate is provided by the simple Bayesian analysis described above. Such a continuum conforming to a single distribution of susceptibilities is observed commonly for artesunate, dihydroartemisinin, artemether, artemisinin, chloroquine, desethylamodiaquine, quinine, quinidine, lumefantrine, piperaquine, pyronaridine and mefloquine. For example, the artesunate-mefloquine combination has been systematically deployed for over twelve years on the north-western border of Thailand. It has been reported in studies of antimalarial susceptibility that the most resistant isolate EC_50 _values were 23.4 ng/mL for artesunate and 197 ng/mL for mefloquine. Reanalysis of the data using the Bayesian approach reduces this to 20.3 (15% less) ng/mL for artesunate and 138 ng/mL (43% less) for mefloquine. When antimalarial drug resistance is reported precise details of the concentration range tested and the analytical procedure used should always be provided.

For those resistance mechanisms in which single mutations confer large reductions in susceptibility, such as the *Pfdhfr *164 mutation for pyrimethamine resistance or *cyt b *268 mutations for atovaquone resistance there will clearly be discontinuous distributions of susceptibility and this method will not be appropriate. This emphasizes the importance of distributional assessments before using this Bayesian approach to analysis. Of course if parasites can be cryopreserved then the resistant parasites dose-response measurements should be repeated with selection of higher concentrations covering the likely EC_50 _region. For freshly assayed isolates this will not be possible as the level of susceptibility is not known before the test. In this case a Bayesian adjustment should be made for extreme values if justified by the distributional assessment.

## Conclusion

Conventional analytical methods for characterizing in-vitro antimalarial drug susceptibility assess each isolate independently and, consequently, overestimate the EC_50 _and EC_90 _for the most resistant isolates. Bayesian methods based on the distribution of EC_50 _values in the whole series offer considerable improvement of the estimate. The method which is proposed here, does not require sophisticated software, nor does it make any assumptions about the dose-response relationship, and it provides more realistic estimates of the most resistant isolates' EC_50 _values

## List of abbreviations

**Emax; **the maximum effect -usually refers to maximum inhibition of growth or substrate uptake or synthesis in an antimalarial drug susceptibility test

**EC**_**50**_; the concentration which results in 50% of the Emax

**EC**_**90 **_; the concentration which results in 90% of the Emax

***γ***; The slope of the linear portion of the usually sigmoid concentration effect relationship

## Authors' contributions

KS, KC, AB, ND, NW contributed to the conceptualization and writing of the paper. AB conducted the in-vitro susceptibility tests. All authors read and approved the final manuscript.

## Appendix I

Useful facts :

1. If f(x) is a density function for random variable x then

F(z) = P(x<z) = ∫−∞zf(x) dx
 MathType@MTEF@5@5@+=feaafiart1ev1aaatCvAUfKttLearuWrP9MDH5MBPbIqV92AaeXatLxBI9gBaebbnrfifHhDYfgasaacH8akY=wiFfYdH8Gipec8Eeeu0xXdbba9frFj0=OqFfea0dXdd9vqai=hGuQ8kuc9pgc9s8qqaq=dirpe0xb9q8qiLsFr0=vr0=vr0dc8meaabaqaciaacaGaaeqabaqabeGadaaakeaadaWdXaqaaiabbAgaMjabcIcaOiabbIha4jabcMcaPiabbccaGiabbsgaKjabbIha4bWcbaGaeyOeI0IaeyOhIukabaGaeeOEaOhaniabgUIiYdaaaa@3AB7@; ∫−∞∞f(x) dx
 MathType@MTEF@5@5@+=feaafiart1ev1aaatCvAUfKttLearuWrP9MDH5MBPbIqV92AaeXatLxBI9gBaebbnrfifHhDYfgasaacH8akY=wiFfYdH8Gipec8Eeeu0xXdbba9frFj0=OqFfea0dXdd9vqai=hGuQ8kuc9pgc9s8qqaq=dirpe0xb9q8qiLsFr0=vr0=vr0dc8meaabaqaciaacaGaaeqabaqabeGadaaakeaadaWdXaqaaiabbAgaMjabcIcaOiabbIha4jabcMcaPiabbccaGiabbsgaKjabbIha4bWcbaGaeyOeI0IaeyOhIukabaGaeyOhIukaniabgUIiYdaaaa@3AAD@;

P(a<x<b) = F(b)-F(a)

where P(x<z) denotes probability that random variable x is less then z.

2. f(x) = 1/(*σ*2
 MathType@MTEF@5@5@+=feaafiart1ev1aaatCvAUfKttLearuWrP9MDH5MBPbIqV92AaeXatLxBI9gBaebbnrfifHhDYfgasaacH8akY=wiFfYdH8Gipec8Eeeu0xXdbba9frFj0=OqFfea0dXdd9vqai=hGuQ8kuc9pgc9s8qqaq=dirpe0xb9q8qiLsFr0=vr0=vr0dc8meaabaqaciaacaGaaeqabaqabeGadaaakeaadaGcaaqaaiabikdaYaWcbeaaaaa@2DB9@*π*)·exp(-(x-*μ*)^2^/2*σ*^2^) density function for N(*μ*, *σ*)

3. f(x) = 1/(2
 MathType@MTEF@5@5@+=feaafiart1ev1aaatCvAUfKttLearuWrP9MDH5MBPbIqV92AaeXatLxBI9gBaebbnrfifHhDYfgasaacH8akY=wiFfYdH8Gipec8Eeeu0xXdbba9frFj0=OqFfea0dXdd9vqai=hGuQ8kuc9pgc9s8qqaq=dirpe0xb9q8qiLsFr0=vr0=vr0dc8meaabaqaciaacaGaaeqabaqabeGadaaakeaadaGcaaqaaiabikdaYaWcbeaaaaa@2DB9@*π*)·exp(-x^2^/2) density function for N(0,1)

4. Φ(x) = 1/(2
 MathType@MTEF@5@5@+=feaafiart1ev1aaatCvAUfKttLearuWrP9MDH5MBPbIqV92AaeXatLxBI9gBaebbnrfifHhDYfgasaacH8akY=wiFfYdH8Gipec8Eeeu0xXdbba9frFj0=OqFfea0dXdd9vqai=hGuQ8kuc9pgc9s8qqaq=dirpe0xb9q8qiLsFr0=vr0=vr0dc8meaabaqaciaacaGaaeqabaqabeGadaaakeaadaGcaaqaaiabikdaYaWcbeaaaaa@2DB9@*π*) ∫−∞zexp(-x2/2) dx
 MathType@MTEF@5@5@+=feaafiart1ev1aaatCvAUfKttLearuWrP9MDH5MBPbIqV92AaeXatLxBI9gBaebbnrfifHhDYfgasaacH8akY=wiFfYdH8Gipec8Eeeu0xXdbba9frFj0=OqFfea0dXdd9vqai=hGuQ8kuc9pgc9s8qqaq=dirpe0xb9q8qiLsFr0=vr0=vr0dc8meaabaqaciaacaGaaeqabaqabeGadaaakeaadaWdXaqaaiabbwgaLjabbIha4jabbchaWjabbIcaOiabb2caTiabbIha4naaCaaaleqabaGaeeOmaidaaOGaee4la8IaeeOmaiJaeeykaKIaeeiiaaIaeeizaqMaeeiEaGhaleaacqGHsislcqGHEisPaeaacqqG6bGEa0Gaey4kIipaaaa@4164@

5. If x is random variable with distribution N(*μ*, *σ*) then

F(x) = Φ((x-*μ*)/*σ*))

## Appendix II

1. Let assume x is N(*μ*, *σ*) with density function f(x) (as in point 2, Appendix I)

2. We want to find median value of x in the interval (a,b)

3. We need to define density function g(x) such that:

g(x)={0if x<=aAf(x)if x in interval(a,b)and∫−∞∞g(x) dx=1.0if x>=b
 MathType@MTEF@5@5@+=feaafiart1ev1aaatCvAUfKttLearuWrP9MDH5MBPbIqV92AaeXatLxBI9gBaebbnrfifHhDYfgasaacH8akY=wiFfYdH8Gipec8Eeeu0xXdbba9frFj0=OqFfea0dXdd9vqai=hGuQ8kuc9pgc9s8qqaq=dirpe0xb9q8qiLsFr0=vr0=vr0dc8meaabaqaciaacaGaaeqabaqabeGadaaakeaacqqGNbWzcqGGOaakcqqG4baEcqGGPaqkcqGH9aqpdaGabeqaauaabeqadqaaaaqaaiabicdaWaqaaiabbMgaPjabbAgaMjabbccaGiabbIha4jabgYda8iabg2da9iabbggaHbqaaaqaaaqaaiabbgeabjabbAgaMjabcIcaOiabbIha4jabcMcaPaqaaiabbMgaPjabbAgaMjabbccaGiabbIha4jabbccaGiabbMgaPjabb6gaUjabbccaGiabbMgaPjabb6gaUjabbsha0jabbwgaLjabbkhaYjabbAha2jabbggaHjabbYgaSjabcIcaOiabbggaHjabcYcaSiabbkgaIjabcMcaPaqaaiabbggaHjabb6gaUjabbsgaKbqaamaapedabaGaee4zaCMaeiikaGIaeeiEaGNaeiykaKIaeeiiaaIaeeizaqMaeeiEaGNaeyypa0JaeGymaeJaeiOla4caleaacqGHsislcqGHEisPaeaacqGHEisPa0Gaey4kIipaaOqaaiabicdaWaqaaiabbMgaPjabbAgaMjabbccaGiabbIha4jabg6da+iabg2da9iabbkgaIbqaaaqaaaaaaiaawUhaaaaa@7985@

∫−∞∞g(x)
 MathType@MTEF@5@5@+=feaafiart1ev1aaatCvAUfKttLearuWrP9MDH5MBPbIqV92AaeXatLxBI9gBaebbnrfifHhDYfgasaacH8akY=wiFfYdH8Gipec8Eeeu0xXdbba9frFj0=OqFfea0dXdd9vqai=hGuQ8kuc9pgc9s8qqaq=dirpe0xb9q8qiLsFr0=vr0=vr0dc8meaabaqaciaacaGaaeqabaqabeGadaaakeaadaWdXaqaaiabbEgaNjabcIcaOiabbIha4jabcMcaPaWcbaGaeyOeI0IaeyOhIukabaGaeyOhIukaniabgUIiYdaaaa@3722@ dx = A (Φ((b-*μ*)/*σ*)) - Φ((a-*μ*)/*σ*))) = 1

Therefore A = 1/(Φ((b-*μ*)/*σ*)) - Φ((a-*μ*)/*σ*))).

4. Median value of x in interval (a,b) is such a z that

G(z) = P(x<z) = ∫−∞zg(x)
 MathType@MTEF@5@5@+=feaafiart1ev1aaatCvAUfKttLearuWrP9MDH5MBPbIqV92AaeXatLxBI9gBaebbnrfifHhDYfgasaacH8akY=wiFfYdH8Gipec8Eeeu0xXdbba9frFj0=OqFfea0dXdd9vqai=hGuQ8kuc9pgc9s8qqaq=dirpe0xb9q8qiLsFr0=vr0=vr0dc8meaabaqaciaacaGaaeqabaqabeGadaaakeaadaWdXaqaaiabbEgaNjabcIcaOiabbIha4jabcMcaPaWcbaGaeyOeI0IaeyOhIukabaGaeeOEaOhaniabgUIiYdaaaa@372C@ dx = 0.5

∫−∞zg(x)
 MathType@MTEF@5@5@+=feaafiart1ev1aaatCvAUfKttLearuWrP9MDH5MBPbIqV92AaeXatLxBI9gBaebbnrfifHhDYfgasaacH8akY=wiFfYdH8Gipec8Eeeu0xXdbba9frFj0=OqFfea0dXdd9vqai=hGuQ8kuc9pgc9s8qqaq=dirpe0xb9q8qiLsFr0=vr0=vr0dc8meaabaqaciaacaGaaeqabaqabeGadaaakeaadaWdXaqaaiabbEgaNjabcIcaOiabbIha4jabcMcaPaWcbaGaeyOeI0IaeyOhIukabaGaeeOEaOhaniabgUIiYdaaaa@372C@ dx = A ∫azf(x)
 MathType@MTEF@5@5@+=feaafiart1ev1aaatCvAUfKttLearuWrP9MDH5MBPbIqV92AaeXatLxBI9gBaebbnrfifHhDYfgasaacH8akY=wiFfYdH8Gipec8Eeeu0xXdbba9frFj0=OqFfea0dXdd9vqai=hGuQ8kuc9pgc9s8qqaq=dirpe0xb9q8qiLsFr0=vr0=vr0dc8meaabaqaciaacaGaaeqabaqabeGadaaakeaadaWdXaqaaiabbAgaMjabcIcaOiabbIha4jabcMcaPaWcbaGaeeyyaegabaGaeeOEaOhaniabgUIiYdaaaa@3615@ dx = A (Φ((z-*μ*)/*σ*)) - Φ((a-*μ*)/*σ*))) =

(Φ((z-*μ*)/*σ*)) - Φ((a-*μ*)/*σ*)))/(Φ((b-*μ*)/*σ*)) - Φ((a-*μ*)/*σ*))) = 0.5

After transformation we get:

Φ((z-*μ*)/*σ*) = (Φ((a-*μ*)/*σ*) + Φ((a-*μ*)/*σ*))/2

and

z = Φ^-1 ^((Φ((a-*μ*)/*σ*) + Φ((a-*μ*)/*σ*))/2)·*σ *+ *μ*
